# Shifts in malaria vector species composition and transmission dynamics along the Kenyan coast over the past 20 years

**DOI:** 10.1186/1475-2875-12-13

**Published:** 2013-01-08

**Authors:** Joseph M Mwangangi, Charles M Mbogo, Benedict O Orindi, Ephantus J Muturi, Janet T Midega, Joseph Nzovu, Hellen Gatakaa, John Githure, Christian Borgemeister, Joseph Keating, John C Beier

**Affiliations:** 1 Kenya Medical Research Institute (KEMRI), Centre for Geographic Medicine Research, Kilifi, Coast, Kenya; 2 Integrated Vector and Disease Management Cluster, International Centre of Insect Physiology and Ecology (icipe), Nairobi, Kenya; 3 Illinois Natural History Survey, University of Illinois, Illinois, Urban-Champaign, USA; 4 Integrated Vector Management Coordinator at the Research Triangle Institute (RTI), Kigali, Rwanda; 5 Department of Global Health Systems and Development, Tulane University School of Public Health and Tropical Medicine, New Orleans, USA; 6 Department of Epidemiology and Public Health, University of Miami Miller School of Medicine, Miami, Florida, USA

## Abstract

**Background:**

Over the past 20 years, numerous studies have investigated the ecology and behaviour of malaria vectors and *Plasmodium falciparum* malaria transmission on the coast of Kenya. Substantial progress has been made to control vector populations and reduce high malaria prevalence and severe disease. The goal of this paper was to examine trends over the past 20 years in *Anopheles* species composition, density, blood-feeding behaviour, and *P. falciparum* sporozoite transmission along the coast of Kenya.

**Methods:**

Using data collected from 1990 to 2010, vector density, species composition, blood-feeding patterns, and malaria transmission intensity was examined along the Kenyan coast. Mosquitoes were identified to species, based on morphological characteristics and DNA extracted from *Anopheles gambiae* for amplification. Using negative binomial generalized estimating equations, mosquito abundance over the period were modelled while adjusting for season. A multiple logistic regression model was used to analyse the sporozoite rates.

**Results:**

Results show that in some areas along the Kenyan coast, *Anopheles arabiensis* and *Anopheles merus* have replaced *An. gambiae sensu stricto (s.s.)* and *Anopheles funestus* as the major mosquito species. Further, there has been a shift from human to animal feeding for both *An. gambiae* sensu lato (s.l.) (99% to 16%) and *An. funestus* (100% to 3%), and *P. falciparum* sporozoite rates have significantly declined over the last 20 years, with the lowest sporozoite rates being observed in 2007 (0.19%) and 2008 (0.34%). There has been, on average, a significant reduction in the abundance of *An. gambiae* s.l. over the years (IRR = 0.94, 95% CI 0.90–0.98), with the density standing at low levels of an average 0.006 mosquitoes/house in the year 2010.

**Conclusion:**

Reductions in the densities of the major malaria vectors and a shift from human to animal feeding have contributed to the decreased burden of malaria along the Kenyan coast. Vector species composition remains heterogeneous but in many areas *An. arabiensis* has replaced *An. gambiae* as the major malaria vector. This has important implications for malaria epidemiology and control given that this vector predominately rests and feeds on humans outdoors. Strategies for vector control need to continue focusing on tools for protecting residents inside houses but additionally employ outdoor control tools because these are essential for further reducing the levels of malaria transmission.

## Background

Fundamental to the development of sound malaria control programmes is an understanding of how malaria transmission intensity affects malaria prevalence, incidence, severe disease, and mortality [1-3]. To gauge levels of malaria control necessary for achieving meaningful public health improvements, it is necessary to quantitatively define the extent to which site-specific malaria transmission indices must be reduced. Effective vector control strategies that negatively impact the components of vectorial capacity can then be developed [4]. Strategies such as indoor spraying with residual insecticides (IRS), sleeping under long-lasting insecticide-treated bed nets (LLINs), larval habitat management (LHM), and the use of repellents and other vector control measures can then be used selectively to reduce levels of transmission.

Several African countries have already scaled up the delivery of key malaria control measures, notably LLINs [5] and the provision of more effective artemisinin-based combination therapy (ACT) for malaria case management [6]. For example, between 2000 and 2008, globally there was a three to 10-fold increase in ownership and use of ITNs among children under five years of age [7] and a 25-fold increase in global procurement of ACT over the last few years [7]. These changes have contributed to the global decline in malaria morbidity and mortality, and may have motivated many countries to begin think-ing through next steps towards malaria elimination [8-10]. Net usage on the coast and throughout Kenya was negligible before 2001. However, LLIN coverage among children five years and below rose from 7% in 2004 to 67% by the end of 2006 [11-13]. This increase in LLIN coverage along with changes in malaria treatment and management, has likely contributed to the steady decline in malaria morbidity and mortality along the Kenyan coast [8-10].

Over the past 20 years, studies on malaria vectors have been conducted along the Kenyan coast. Initially studies focused on malaria transmission dynamics and high incidence of severe malaria [14-17], but surprisingly found lower transmission than in western Kenya – providing evidence that high incidence of severe malaria can occur even at relatively low intensities of transmission [16,17]. These studies were proceeded by investigations of mosquito blood-feeding behaviour [15,18], vector distribution patterns [19,20], spatial-temporal variations in malaria prevalence and intensity of transmission [20], vector population genetics [21], community-based vector control [22,23], malaria vector control [24-26] and larval ecology of malaria vectors [27-29].

In the light of on-going malaria control interventions and the recent downward trend of malaria prevalence along the Kenyan coast, accurate knowledge of how these interventions have altered the ecology of major vectors and the risk of malaria transmission will facilitate allocation of vector control efforts where they are most needed. The aim of this paper is to examine trends in *Anopheles* species composition, densities, and blood-feeding behaviour over a 20-year period, between 1990 and 2010. The results are discussed in terms of malaria transmission dynamics and vector control to explore what more is needed to further interrupt transmission on the coast of Kenya.

## Methods

### Study area

**Figure 1:**
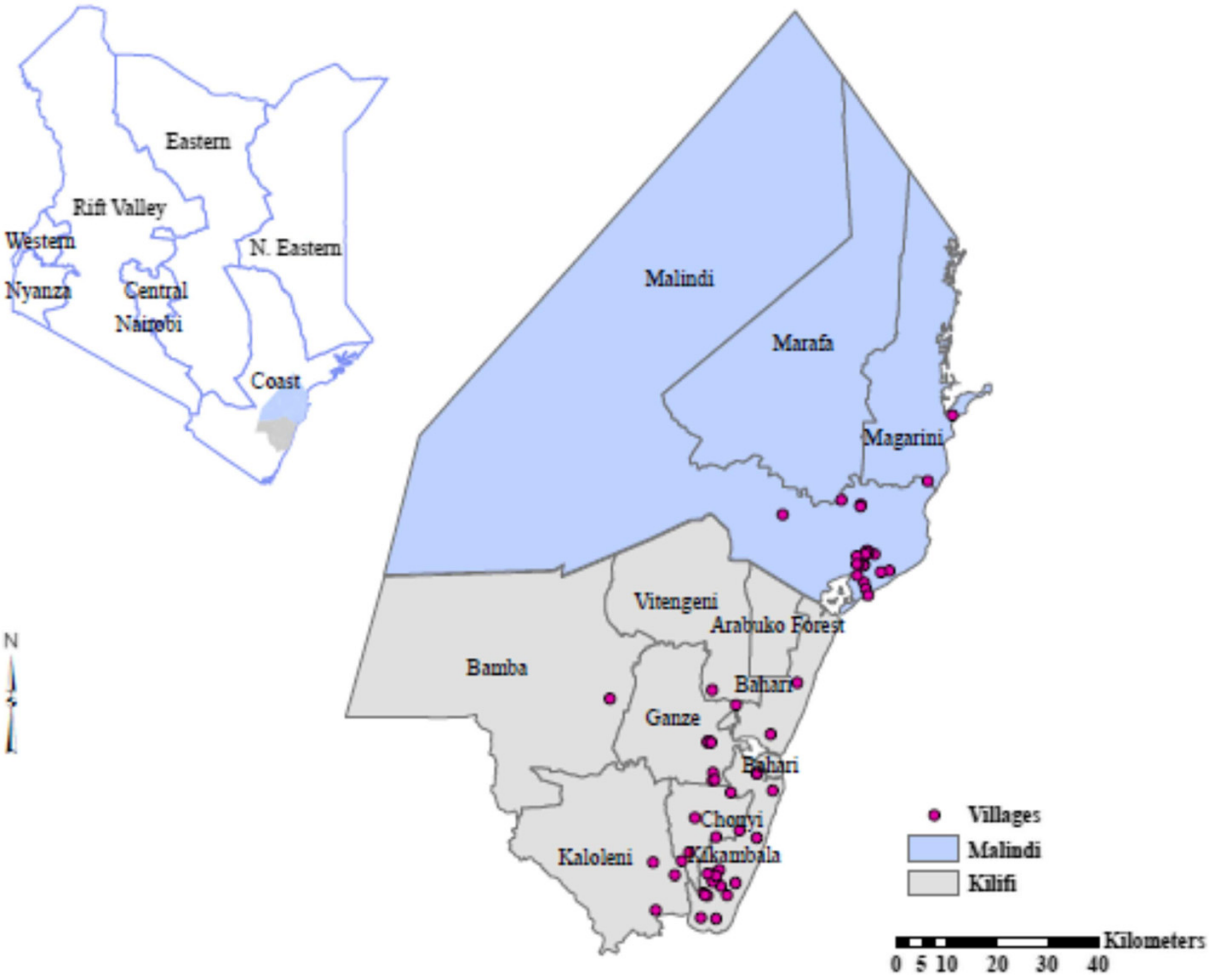
Map showing the locations of villages sampled in Kilifi and Malindi Districts.

Studies from 1990 to 2010 were conducted in 49 villages located in Malindi and Kilifi Districts on the Kenyan coast (Figure 1). The data collected during these periods was standardized to show mean indoor-resting mosquito densities over different periods. The study areas have been described in detail elsewhere [15,19-22,24-27,29-34]. Briefly, the coastal plain is made up of dense forest, savannah type vegetation, seasonal swamps, dry thorn bush, and a number of plantations interspersed with uncultivated land. Altitudes range from 0 to 400 m above sea level. Sisal, coconut, and cashew nut plantations are extensive along the coast, although subsistence farming is practiced throughout the coastal area. The houses in rural areas of coastal Kenya mainly consist of framed poles and branches from top to bottom covered with grass. Mud is often used to support the upper structure, while palm leaves often replace grass as roofing material. Many households keep goats, chickens, and cattle as domestic animals.

Coastal Kenya has two rainy seasons: the long rains occur between April and July and the short rains occur between October and November. Mean annual precipitation ranges from 750 to 1,200 mm [34]. Over the past 20 years, people living in near proximity to the Sabaki and Jaribuni rivers have started small-scale irrigated agriculture. The Kenyan Government, through the Economic Stimulus Programme and Vision 2030 Strategies has also continued to develop the Lango Baya Irrigation Scheme in Malindi. Horticultural crops grown under these irrigation activities include kale, hot pepper, okra, green maize, brinjals (aubergine) and tomatoes. The agronomic practices in the irrigation system use pesticides, mainly of the class organophosphates.

### Mosquito sampling

Several methods were used for entomological sampling, including Centers for Disease Control and prevention (CDC) light traps, pyrethrum spray collection (PSC), human landing catches, and manual aspiration for collecting day resting mosquitoes indoors (DRI) [15,18,20,21,25,32]. The mosquitoes were expressed in densities (mean mosquitoes per house or trap) for the analyses. To overcome some of the limitation in data collections in which the relative use of each method was not the same during each year; the sampling was categorized according to the rainfall pattern along the Kenyan coast. This grouped each year in 4 categories namely long wet season (April to July), short wet season (October and November), long dry season (December to March) and short dry season (August and September).

### Mosquito identification

Mosquitoes were identified to species based on morphological characteristics [35]. Females were further classified as unfed, blood-fed, semi-gravid or gravid [36]. Genomic DNA was extracted from the legs and wings of a proportion of females in the *Anopheles gambiae* complex using the methods of Collins *et al*[37,38] and amplified using specific diagnostic primers for *An. gambiae s.s., Anopheles arabiensis, Anopheles quadriannulatus* and *Anopheles merus* according to previously described methods [39,40]. *Anopheles funestus*, which is a complex of nine sibling species [41,42], was not identified to sibling species level.

### Circumsporozoite protein and blood meal ELISA

The mosquitoes were cut transversely between the thorax and abdomen. The heads and thoraces of anopheline mosquitoes were tested using a *Plasmodium falciparum* sporozoite enzyme linked immunosorbent assay (ELISA) [43-45]. The fully blood-fed abdomens were tested for host sources of blood by ELISA [46]. Test samples were visually assessed for positivity [47].

### Statistical analysis

The analyses were performed using STATA v10.1 (StataCorp, College Station, TX, USA). Both descriptive and inferential analyses were considered. To study the mosquito abundance over the 20-year period while adjusting for season and pre-post LLIN coverage scale-up, a negative binomial generalized estimating equations (GEE) model, assuming exchangeable working correlation, taking calendar year, pre-post scale-up (coded 1 if year is after 2003, and 0 otherwise) and season (wet or dry) as the covariates with household as the cluster was used [48,49]. In this case, wet season comprised the months April, May, June, October, and November. Because of limiting software capability, a standard negative binomial model was used to initially obtain a maximum likelihood value for the ancillary parameter. The same model was fitted separately for *An. gambiae* and *An. funestus*. Mosquito collections were presented as densities in each sampling village. To study the sporozoite rate over the period while controlling for season, a multiple logistic regression model was fitted, but with season redefined into four categories: long wet season, short wet season, long dry season and short dry season. This is because field sampling was done in different months over the year. Odds ratios (OR) were computed for each season in comparison to the long wet season (April to July), which had the highest number of *P. falciparum* sporozoite positive mosquitoes.

The *P. falciparum* sporozoite rates were calculated as the number of mosquitoes that tested positive by ELISA divided by the total number of mosquitoes tested. The proportions of mosquitoes feeding on a given host were compared using Chi- square and/or Fisher’s exact test. These analyses did not assume spatial dependence among the villages. All tests were performed at the 5% level.

## Results

### Vector abundance

**Figure 2:**
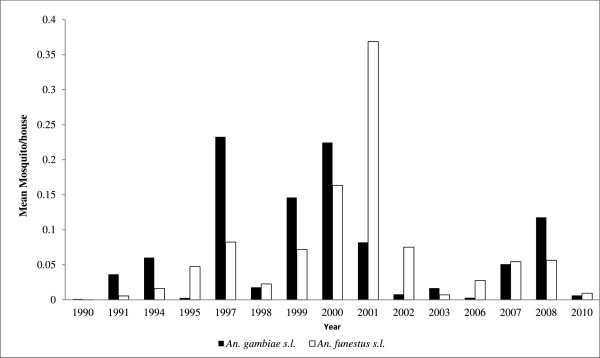
**The *Anopheles gambiae *s.l. and *An. funestus *s.l. mean densities from 1990 to 2010.**

Of 33,529 mosquitoes collected, 18,194 (54.3%: 96% CI 53.7–56.8) were *An. gambiae* and the rest *An. funestus*. This proportion was significantly greater in the wet season (63.6%) than in dry season (45.1%; *X*^*2*^ = 1,158.3, df = 1, p < 0.001). Table 1 shows the distribution of the two species by year and season. Apart from recently in 2010, the densities of *An. funestus s.l.* only exceeded *An. gambiae s.l.* in 2001 and 2002 (Figure 2). Figure 2 and Table 1 show that the densities of both mosquito species increased between 1990 and 2001, thereafter the densities of both *An. gambiae* s.l. and *An. funestus* declined. After controlling for the other two factors, there was, on average, a significant 6% reduction the chance of observing an *An. gambiae s.l.* (IRR = 0.94, 95% CI 0.90–0.98), but a significant 32% increase in the chance of observing an *An. funestus s.l.* (IRR = 1.32, 95% CI 1.25–1.39) for a unit increase in time/year. Season was also significantly associated with the abundance of both *An. gambiae* and *An. funestus s.l.* The abundance of *An. gambiae s.l.* was significantly lower in dry season as compared to wet season (IRR = 0.51, 95% CI 0.49–0.53) after adjusting for year and LLIN scale-up. *Anopheles funestus* were, however, more abundant in dry season than wet season (IRR = 1.08, 95% CI 0.90–1.30), although not significantly so. A significant reduction of both species in the post LLIN distribution scale-up period was observed (*An. gambiae s.l.*: IRR = 0.08, 95% CI 0.04–0.17; *An. funestus s.l.*: IRR = 0.10, 95% CI 0.04–0.24). The ancillary parameter was 8.74 and 14.58 for *An. gambiae* and *An. funestus* models, respectively.

**Table 1:** **Observed percent distribution of *Anopheles gambiae *s.l. and *Anopheles funestus *s.l. by year and season**

**Variables**		**# Observed**	***% An. gambiae *s.l.**	***% An. funestus *s.l.**
All		33,529	54.3	45.7
Covariates				
*Year*				
	1990	22	95.5	4.5
	1991	673	91.8	8.2
	1994	1,236	86.6	13.4
	1995	530	9.1	90.9
	1997	4,970	83.2	16.8
	1998	549	57.9	42.1
	1999	3,325	78.0	22.0
	2000	5,646	70.6	29.4
	2001	5,193	27.9	72.1
	2002	901	15.3	84.7
	2003	367	80.4	19.6
	2006	330	16.1	83.9
	2007	4,773	24.3	75.7
	2008	4,700	44.6	55.4
	2010	314	67.5	32.5
*Pre-post scale-up*				
	pre	23,412	62.7	37.3
	post	10,117	34.8	65.2
*Season*				
	Wet	16,658	63.6	36.4
	Dry	16,871	45.1	54.9

### Shift in *Anopheles gambiae* s.l. sibling species composition over time

Of the four *An. gambiae* complex sibling species identified, *An. gambiae* s.s., which was the dominant indoor-resting mosquito species between 1997 and 1998 accounting for 78.89%, has been declining over the years significantly to very low levels and sometimes undetectable levels in the study sites, especially between 2007 and 2008. On the other hand, *An. arabiensis* has seen an upward trend from 14.84% between 1997 and 1998 to being the dominant *An. gambiae* complex sibling species in late 2000 (Table 2). *Anopheles merus* increased between 2002 and 2003 but declined after 2007. *Anopheles quadriannulatus*, a member of the *An. gambiae* complex, that was not known to occur along the Kenyan coast, was found in 2007, accounting for nearly 2% of the sample (Table 2).

**Table 2:** **Changes in proportion of *Anopheles gambiae *s.l. sibling composition over the years**

**Period**	**Number tested**	***% An. gambiae ss***	***% An. arabiensis***	***% An. merus***	***% An. quadriannulatus***
1990 - 1995*	-	-	-	-	***-***
1997 - 1998	1,388	78.89	14.84	6.27	0.00
2002 - 2003	803	45.70	5.48	48.82	0.00
2007 - 2008	595	0.00	93.02	5.00	1.97

*An. gambiae s.l. mosquitoes not tested for sibling species.

### *Plasmodium falciparum* sporozoite rates

A total of 33,383 mosquitoes (*An. gambiae* s.l. and *An. funestus*) were tested for *P. falciparum* sporozoites. Data for the years 1997 to 2008 was used. The overall sporozoite rate was 4.6%. High sporozoite rates were observed in 1997 (5.94%), 2000 (7.96%), 2001 (6.83%), and 2003 (8.65%), which had the highest rate. There was a significant association between *P. falciparum* sporozoite positivity and year. Compared to 1997 and after controlling for season, there was significantly lower sporozoite rate in 2002 (OR = 0.68, 95%CI 0.51–0.91), 2007 (OR = 0.03, 95% CI 0.01–0.10), and 2008 (OR = 0.06, 95%CI 0.03–0.11). The odds of a mosquito being positive were also lower in 1998, but not significantly so (OR = 0.65, 95% CI 0.35–1.19). After controlling for year, season was also found to be significantly associated with sporozoite positivity. Compared to long wet season, the odds of being positive were significantly high during the long dry (OR = 1.23, 95% CI 1.02–1.49) and short dry seasons (OR = 1.14, 95% CI 1.00–1.29) (Table 3).

**Table 3:** *Plasmodium falciparum *sporozoite rates and logistic regression model results*

**Variables**		**# Tested**	**% Positive**	**OR**	**95% CI**	**P value**
All		33,383	4.60			
Explanatory						
*Year*						
	1997	1,027	5.94	1		
	1998	335	3.88	0.65	(0.35-1.19)	0.162
	2000	5,555	7.96	**1.50**	**(1.12-2.01)**	0.006
	2001	4,521	6.83	1.24	(0.92-1.67)	0.154
	2002	17,210	3.83	**0.68**	**(0.51-0.91)**	0.010
	2003	451	8.65	1.38	(0.90-2.12)	0.141
	2007	1,601	0.19	**0.03**	**(0.01-0.10)**	<0.001
	2008	2,683	0.34	**0.06**	**(0.03-0.11)**	<0.001
*Season*						
	Long Wet	18,214	4.62	1		
	Short Wet	3,206	4.09	1.00	(0.81-1.22)	0.968
	Long Dry	4,095	4.71	**1.23**	**(1.02-1.49)**	0.034
	Short Dry	7,868	4.70	**1.14**	**(1.00-1.29)**	0.044

* Bold = significant at 5% level; OR = odds ratio; CI = confidence interval.

Further analyses indicated that although *P. falciparum* infectiousness by *Anopheles* mosquitoes as indicated by positive sporozoite rates was found to be taking place throughout the four seasons, there was seasonal variability in sporozoite rates during each year (Table 4). The highest sporozoite rate observed during this period was 23.81% during the short dry season in 1998.

**Table 4:** The number of mosquitoes tested in each season and sporozoite rates (in parentheses)

	**Total *Anopheles *mosquitoes tested**			
**Year**	**Long Wet**	**Short Wet**	**Long Dry**	**Short Dry**
1997	47 (2.13)	315 (2.86)	511(5.68)	154 (14.29)
1998	7 (0.00)	139 (3.60)	168 (1.79)	21 (23.81)
2000	3,150 (6.29)	1,157 (9.16)	207 (5.80)	1,041 (12.10)
2001	2,629 (8.18)	273 (3.30)	604 (6.62)	1,015 (4.43)
2002	11,573 (3.66)	a*	982 (6.62)	4,655 (3.65)
2003	a*	a*	451 (8.65)	a*
2007	37 (0.00)	709 (0.14)	515 (0.39)	340 (0.00)
2008	771 (0.39)	613 (0.16)	659 (0.46)	642 (0.31)

Legend: a* No *Anopheles* mosquitoes tested for sporozoites.

### *Anopheles* host feeding patterns over time

Table 5 presents a summary of the feeding patterns over time. There has been a significant reduction in human blood index (HBI) from 99% and 100% to 16% and 3% for *An. gambiae* s.l*.* and *An. funestus,* respectively, between 1997 and 2008. These mosquitoes have switched to primarily feeding on bovines. Other meal sources included, bovine, goat, donkey, human-bovine, and chicken – although only *An. gambiae* was noted to feed on chicken but, again, this was only in 1997.

**Table 5:** **Host-feeding patterns of *Anopheles funestus *and *Anopheles gambiae *s.l. between 1997 and 2008 (parenthesis shows proportion)**

**Species**	**Year**	**Human**	**Bovine**	**Goat**	**Donkey**	**Human-Bovine**	**Chicken**
*An. funestus*	1997	148 (1.00)	0	0	0	0	0
	1998	35 (0.97)	0	1 (0.03)	0	0	0
	2002	1,458 (0.85)	249 (0.15)	0	0	0	0
	2007	7 (0.05)	113 (0.80)	20 (0.14)	1 (0.01)	1 (0.01)	0
	2008	2 (0.03)	47 (0.80)	8 (0.14)	0	2 (0.03)	0
*An. gambiae* s.l.	1997	826 (0.99)	0	3 (0.004)	0	1 (0.001)	2 (0.002)
	1998	283 (0.99)	0	3 (0.01)	0	1 (0.003)	0
	2002	245 (0.80)	62 (0.20)	0	0	0	0
	2007	38 (0.14)	202 (0.75)	27 (0.10)	0	2 (0.01)	0
	2008	41 (0.16)	187 (0.72)	28 (0.11)	0	4 (0.02)	0

## Conclusion

The present study illustrates marked changes in densities of *An. gambiae* s.l. and *An. funestus*. *Anopheles gambiae* s.l. decreased markedly between 1990 and 2008 while *An. funestus* increased between 1990 and 2002 before decreasing thereafter. Similar trends have been reported in western Kenya [50] and in Tanzania [51,52], even in areas where LLINs are not widely used [51]. It appears that multiple factors, including ongoing vector control interventions (e g, widespread use of LLINs, indoor residual spraying), improvements in house constructions [53,54], and human-mediated ecological disturbances [55] may have contributed to the observed decline in mosquito density. The Kenya coast in areas along the Sabaki River, such as Lango Baya, Chakama and Burangi, has seen an increase in irrigated agricultural activities that have a direct influence in vector densities and behaviour.

The primary vectors of malaria on the Kenyan coast have shifted from human to animal feeding; this has also coincided with significant reductions in sporozoite rates. Along the Kenyan coast there was mass distribution of LLINs in 2006 and in 2012 there was a mass LLIN distribution to achieve universal coverage. Reductions in mosquito density may be attributed to ongoing mass distribution of LLINs in the study area [5,11]. Long-term use of LLINs impacts malaria vectors by killing/repelling host-seeking mosquitoes, diverting host-seeking mosquitoes to non-human hosts and/or inducing shifts from indoor to outdoor feeding. The observed reduction in vector density and shifts from human to animal feeding suggest that all these mechanisms have contributed to the declining malaria prevalence. Animals are dead-end hosts for human malaria parasites and may have contributed to low sporozoite rates by reducing the probability of human-vector contact.

The observed decrease in *An. gambiae s.s* populations and the coinciding increase in *An. arabiensis* populations may be attributed to differences in their ecology. *Anopheles gambiae s.s.* is anthropophilic, endophagic and endophilic and its frequent contact with LLINs may have contributed to its downward trend. Conversely, *An. arabiensis* exhibits a combination of exophilic and partial zoophilic tendencies, which may have reduced its contact with LLINs promoting its upward trend [50,56-58]. Some studies along the Kenya coast indicate that *An. gambiae s.s.* may be found occurring in some focal areas which have been characterized as malaria hotspots in Kilifi District [59]. Understanding the ecology of *An. gambiae* and other malaria vectors in these hotspots will be a step closer towards the achievement of malaria-specific Millennium Development Goals [60-63].

In the current study, members of the *An. funestus* complex were not identified to species due to logistic difficulties. However, given the observed shift from human to animal feeding, it is likely that *Anopheles parensis,* a predominantly zoophilic member of *An. funestus* complex, has replaced the highly anthropophilic *An. funestus s.s*. as the most dominant species of this complex [41,42,64]. Currently molecular capability for analysis of sibling species of *An. funestus*[41,64] is well developed; consequently further studies are needed to assess the distribution and abundance of sibling species of the *An. funestus* complex along the Kenyan coast.

In conclusion, the results seen on the Kenyan coast show a downward trend in key entomological indices, which may help explain the reduction seen in malaria prevalence and incidence. The changes in the composition of *An. gambiae* complex have undoubtedly important implications for the epidemiology and strategies for control of malaria in the study area. With increase of *An. arabiensis* populations along the Kenya coast, mosquito control strategies should incorporate both indoor and outdoor control tools, which will help in significantly reducing the levels of malaria transmission. Scale up of LLINs to universal coverage, coupled with larval habitat management strategies, stakeholder involvement and community engagement packaged in integrated vector management (IVM) strategy, would be ideal to significantly reduce indoor and outdoor resting vectors [65-67].

## Competing interests

The authors declare that they have no competing interests.

## Authors’ contributions

JMM, BOO, JTM, JN, and HG conducted all data mining, extraction and preparations and further provided scientific guidance in data cleaning, analysis and manuscript preparation. CMM, EJM, JG and CB, offered scientific guidance in data analysis and manuscript preparation. JK and JCB provided overall supervision of the study and preparation of manuscript. All authors actively contributed to the interpretation of the findings and development of the final manuscript and approved the final manuscript.

## References

[B1] Alles HK Mendis K Carter R Malaria mortality rates in South Asia and in Africa: implications for malaria control Parasitol Today 1998 14 369 375 10.1016/S0169-4758(98)01296-4 17040819

[B2] Greenwood B Marsh K Snow R Why do some African children develop severe malaria? Parasitol Today 1991 7 277 281 10.1016/0169-4758(91)90096-7 15463389

[B3] Marsh K Malaria - a neglected disease? Parasitology 1992 104 S53 S69 10.1017/S0031182000075247 1589300

[B4] Eisele TP Steketee RW African malaria control programs deliver ITNs and achieve what the clinical trials predicted PLoS Med 2011 8 e1001088 1001010.1001371/journal.pmed.1001088 21909247 10.1371/journal.pmed.1001088 PMC3167796

[B5] Noor AM Mutheu JJ Tatem AJ Hay SI Snow RW Insecticide treated net coverage in Africa: mapping progress in 2000-07 Lancet 2009 373 58 67 10.1016/S0140-6736(08)61596-2 19019422 PMC2652031

[B6] RBM The global malaria action plan for a free malaria world 2008 Geneva: Roll Back Malaria Partnership, World Health Organization

[B7] UNICEF Malaria and children: Progress in intervention coverage 2009 New York: United Nations Children’s Fund

[B8] Okiro EA Alegana VA Noor AM Mutheu JJ Juma E Snow RW Malaria paediatric hospitalization between 1999 and 2008 across Kenya BMC Med 2009 7 75 10.1186/1741-7015-7-75 20003178 PMC2802588

[B9] Okiro EA Hay SI Gikandi PW Sharif SK Noor AM Peshu N Marsh K Snow RW The decline in paediatric malaria admissions on the coast of Kenya Malar J 2007 6 151 10.1186/1475-2875-6-151 18005422 PMC2194691

[B10] O’Mearaa WP Bejon P Mwangi TW Okiro EA Peshu N Snow RW Newton CRJC Marsh K Effect of a fall in malaria transmission on morbidity and mortality in Kilifi Kenya. Lancet 2008 372 1555 1562 10.1016/S0140-6736(08)61655-4 PMC2607008 18984188

[B11] Noor AM Amin AA Akhwale WS Snow RW Increasing Coverage and Decreasing Inequity in Insecticide-Treated Bed Net Use among Rural Kenyan children PLoS Med 2007 4 e255 10.1371/journal.pmed.0040255 17713981 PMC1949846

[B12] Noor AM Moloney G Borle M Fegan GW Shewchuk T Snow RW The use of mosquito nets and the prevalence of Plasmodium falciparum infection in rural south central Somalia PLoS One 2008 3 e2081 10.1371/journal.pone.0002081 18461178 PMC2362695

[B13] Noor AM Mutheu JJ Tatem AJ Hay SI Snow RW Insecticide-treated net coverage in Africa: mapping progress in 2000-07 Lancet 2008 10.1016/S0140-6736(08)61596-2 PMC2652031 19019422

[B14] Kabiru EW Mbogo CM Muiruri SK Ouma JH Githure JI Beier JC Sporozoite loads of naturally infected Anopheles in Kilifi District, Kenya J Am Mosq Control Assoc 1997 13 259 262 9383768

[B15] Mbogo CNM Kabiru EW Muiruri SK Nzovu JM Ouma JH Githure JI Beier JC Bloodfeeding behaviour of Anopheles gambiae s.l. and Anopheles funestus in Kilifi district, Kenya J Am Mosq Control Assoc 1993 9 225 227 8350080

[B16] Mbogo CNM Snow RW Kabiru EW Ouma JH Githure JI Marsh K Beier JC Low-level Plasmodium falciparum transmission and the incidence of severe malaria infections on the Kenyan coast AmJTrop Med Hyg 1993 49 245 253 10.4269/ajtmh.1993.49.245 8357087

[B17] Mbogo CNM Snow RW Khamala CPM Kabiru EW Ouma JH Githure JI Marsh K Beier JC Relationships between Plasmodium falciparum transmission by vector populations and the incidence of severe disease at nine sites on the Kenyan coast Am J Trop Hyg 1995 52 201 206 10.4269/ajtmh.1995.52.201 7694959

[B18] Mwangangi JM Mbogo CM Nzovu JG Githure JI Yan G Beier JC Blood meal analysis for anopheline mosquitoes sampled along the Kenyan coast J Am Mosq Control Assoc 2003 19 371 375 14710739

[B19] Keating J Mbogo CM Mwangangi J Nzovu JG Gu W Regens JL Yan G Githure JI Beier JC Anopheles gambiae s.l. and Anopheles funestus mosquito distributions at 30 villages along the Kenyan Coast J Med Entomol 2005 42 241 246 10.1603/0022-2585(2005)042[0241:AGSAAF]2.0.CO;2 15962770 PMC2673524

[B20] Mbogo CM Mwangangi JM Nzovu J Gu W Yan G Gunter J Swalm C Keating J Regens JL Shililu JI Githure JI Beier JC Spatial and temporal heterogeneity of Anopheles mosquitoes and Plasmodium falciparum transmission along the Kenyan coast AmJTrop Med Hyg 2003 68 734 742 12887036

[B21] Midega JT Mbogo CM Mwambi H Wilson MD Ojwang G Mwangangi JM Nzovu JG Githure JI Yan G Beier JC Estimating population density, dispersal and survival for Anopheles gambiae and Anopheles funestus along the Kenyan Coast using mark-release-recapture methods J Med Entomol 2007 44 923 929 10.1603/0022-2585(2007)44[923:EDASOA]2.0.CO;2 18047189 PMC2705338

[B22] Kibe LW Mbogo CM Keating J Molyneux S Githure JI Beier JC Community based vector control in Malindi, Kenya Afr Health Sci 2006 6 240 246 17604514 10.5555/afhs.2006.6.4.240 PMC1832065

[B23] Keating J Macintyre K Mbogo CM Githure JI Beier JC Self-reported malaria and mosquito avoidance in relation to household risk factors in a Kenyan coastal city J Biosoc Sci 2005 37 761 771 10.1017/S0021932005007182 16221324 PMC2705334

[B24] Macintyre K Keating J Sosler S Kibe L Mbogo CM Githeko AK Beier JC Examining the determinants of mosquito-avoidance practices in two Kenyan cities Malar J 2002 1 14 10.1186/1475-2875-1-14 12495438 PMC149385

[B25] Mbogo CM Baya NM Ofulla AVO Githure JI Snow RW The impact of permethrin-impregnated bednets on malaria vectors of the Kenyan coast Med Vet Entomol 1996 10 251 259 10.1111/j.1365-2915.1996.tb00739.x 8887336

[B26] Mwangangi JM Kahindi SC Kibe LW Nzovu JG Luethy P Githure JI Mbogo CM Wide-scale application of Bti/Bs biolarvicide in different aquatic habitat types in urban and peri-urban Malindi, Kenya Parasitol Res 2011 108 1355 1363 10.1007/s00436-010-2029-1 20730445

[B27] Keating J Macintyre K Mbogo CM Githure JI Beier JC Characterization of potential larval habitats for Anopheles mosquitoes in relation to urban land-use in Malindi, Kenya Int J Health Geog 2004 3 9 10.1186/1476-072X-3-9 PMC419712 15125778

[B28] Mwangangi J Mbogo C Nzovu JG Muturi EJ Githure JI Minakawa N Yan G Novak RJ Beier JC Spatial distribution and habitat characterisation of Anopheles larvae along the Kenyan coast J Vector Borne Dis 2007 44 44 51 17378216 PMC2731850

[B29] Mwangangi JM Mbogo CM Muturi EJ Kabiru EW Githure JI Novak RJ Beier JC The influence of biological and physicochemical characteristics of larval habitat on the body size of Anopheles gambiae (Diptera: Culicidae) mosquitoes J Vector Borne Dis 2007 44 121 126 PMC2705333 17722866

[B30] Kahindi S Midega JT Mwangangi JM Kibe L Nzovu J Luethy P Githure J Mbogo C The efficacy of Vectobac DT and Culinexcombi against mosquito larvae in unused swimming pools in Malindi, Kenya J Am Mosq Control Assoc 2008 24 538 542 10.2987/5734.1 19181062

[B31] Keating J Macintyre K Mbogo CM Githeko A Regens JL Swalm C Ndenga B Steinberg LJ Kibe L Githure JI Beier JC A geographic sampling strategy for studying relationships between human activity and malaria vectors in urban Africa AmJTrop Med Hyg 2003 68 357 365 12685645

[B32] Mbogo CMN Glass GE Forster D Kabiru EW Githure JI Ouma JH Beier JC Evaluation of light traps for sampling anopheline mosquitoes in Kilifi, Kenya J Am Mosq Control Assoc 1993 9 260 263 8245934

[B33] Muturi JE Mbogo C Mwangangi J Ng’ang’a Z Kabiru E Mwandawiro C Beier JC Concomitant infections of Plasmodium falciparum and Wuchereria bancrofti on the Kenyan coast Filaria J 2006 5 8 10.1186/1475-2883-5-8 16723020 PMC1513226

[B34] Mwangangi JM Midega J Kahindi S Njoroge L Nzovu J Githure J Mbogo CM Beier JC Mosquito species abundance and diversity in Malindi, Kenya and their potential implication in pathogen transmission Parasitol Res 2012 110 61 71 10.1007/s00436-011-2449-6 21626425

[B35] Gillies MT Coetzee M A supplement to anophelinae of Africa south of Sahara (Afro-tropical region) Publication of the South Africa Institute of Medical Research 1987 55 1 143

[B36] WHO Manual on practical entomology in Malaria. Part II. Methods and techniques 1975 Geneva: World Health Organization Offset Publication No. 13

[B37] Collins FH Mehaffey PC Rasmussen MO AD B-B Odera JS Finnerty V Comparison of DNA-probe and isoenzyme methods for differentiating Anopheles gambiae and Anopheles arabiensis (Diptera: culicidae) J Med Entomol 1988 25 6 20 10.1093/jmedent/25.2.116 3280799

[B38] Collins FH Petrarca V Mpofu S Brandling-Benneth AD Were JB Rasmusssen MO Finerty V Comparison of DNA-Probe and cytogenetic methods for identifying field collected Anopheles gambiae complex mosquitoes AmJTrop Med Hyg 1988 39 545 550 10.4269/ajtmh.1988.39.545 3207175

[B39] Paskewitz SM Collins FH Use of the polymerase chain reaction to identify mosquito species of the Anopheles gambiae complex Med Vet Entomol 1990 4 367 373 10.1111/j.1365-2915.1990.tb00453.x 2133004

[B40] Scott JA Brodgon WG Collins FH Identification of single specimens of Anopheles gambiae complex by polymerase chain reaction AmJTrop Med Hyg 1993 49 520 529 10.4269/ajtmh.1993.49.520 8214283

[B41] Kamau L Munyekenye GO Koekemoer LL Hunt RH Coetzee M A survey of the Anopheles funestus (Diptera: Culicidae) group of mosquitoes from 10 sites in Kenya with special emphasis on population genetic structure based on chromosomal inversion karyotypes J Med Entomol 2003 40 664 671 10.1603/0022-2585-40.5.664 14596280

[B42] Koekemoer LL Coetzee M Hunt RH Hpall endonuclease distinguishes between two species in the Anopheles funestus group Insect Mol Biol 1998 7 1 5 10.1046/j.1365-2583.1998.71046.x 9662477

[B43] Beier JC Perkins PV Wirtz RA Whitmire RE Mugambi M Hockmeyer WT Field evaluation of an enzyme-linked immunosorbent assay (ELISA) for Plasmodium falciparum sporozoite detection in anopheline mosquitoes from Kenya AmJTrop Med Hyg 1987 36 459 468 10.4269/ajtmh.1987.36.459 3555134

[B44] Wirtz RA Burkot TR Detection of malarial parasites in mosquitoes Adv Dis Vect Res 1991 8 77 106 10.1007/978-1-4612-3110-3_4

[B45] Wirtz RA Zavala F Charoenvit Y Campbell GH Burkot TR Schneider I Esser KM Beaudoin RL Andre RG Comperative testing of Plasmodium falciparum Circumsporozoite antibody Bull World Health Organ 1987 65 39 45 3555879 PMC2490858

[B46] Beier JC Asiago CM Onyango FK Koros JK ELISA absorbance cut-off method affects malaria sporozoite rate determination in wild Afrotropical Anopheles Med Vet Entomol 1988 2 259 264 10.1111/j.1365-2915.1988.tb00193.x 2980182

[B47] Beier JC Koros J Visual assessment of sporozoite and blood meal ELISA samples in malaria field studies J Med Entomol 1991 28 805 808 1770515 10.1093/jmedent/28.6.805

[B48] Liang KY Zeger S Longitudinal data analysis using generalized linear models Biometrica 1986 73 13 22 10.1093/biomet/73.1.13

[B49] Molenberghs G Verbeke G Models for Discrete Longitudinal Data 2005 New York: Springer

[B50] Bayoh NM Mathias DK Odiere MR Mutuku FM Kamau L Gimnig JE Vulule JM Hawley WA Hamel MJ Walker EW Anopheles gambiae: historical population decline associated with regional distribution of insecticide-treated bed nets in western Nyanza Province, Kenya Malar J 2010 9 62 10.1186/1475-2875-9-62 20187956 PMC2838909

[B51] Meyrowitsch DW Pedersen EM Alifrangis M Scheike TH Malecela MN Magesa SM Derua YA Rwegoshora RT Michael E Simonsen PE Is the current decline in malaria burden in sub-Saharan Africa due to a decrease in vector population? Malar J 2011 10 188 10.1186/1475-2875-10-188 21752273 PMC3160426

[B52] Derua YA Alifrangis M Hosea KM Meyrowitsch DW Magesa SM Pedersen EM Simonsen PE Change in composition of the Anopheles gambiae complex and its possible implications for the transmission of malaria and lymphatic filariasis in north-eastern Tanzania Malar J 2012 11 188 10.1186/1475-2875-11-188 22681999 PMC3469399

[B53] Konradsen F Amerasinghe P van der Hoek W Amerasinghe F Perera D Piyaratne M Strong association between house characteristics and malaria vectors in Sri Lanka AmJTrop Med Hyg 2003 68 177 181 12641408

[B54] Atieli H Menya D Githeko A Scott T House design modifications reduce indoor resting malaria vector densities in rice irrigation scheme area in western Kenya Malar J 2009 8 108 10.1186/1475-2875-8-108 19454025 PMC2688520

[B55] Munga S Yakob L Mushinzimana E Zhou G Ouna T Minakawa N Githeko A Yan G Land use and land cover changes and spatiotemporal dynamics of anopheline larval habitats during a four-year period in a highland community of Africa AmJTrop Med Hyg 2009 81 1079 1084 10.4269/ajtmh.2009.09-0156 PMC3726196 19996440

[B56] Russell TL Lwetoijera DW Maliti D Chipwaza B Kihonda J Charlwood D Smith TA Lengeler C Mwanyangala MA Nathan R Knols BGJ Takken W Killeen GF Impact of promoting longer-lasting insecticide treatment of bed nets upon malaria transmission in a rural Tanzanian setting with pre-existing high coverage of untreated nets Malar J 2010 9 187 10.1186/1475-2875-9-187 20579399 PMC2902500

[B57] Mendis C Jacobsen JL Gamage-mendis A Dgedge M Thompson R Cuamba N Barreto J Begtrup K Sinden RE Hogh B Anopheles arabiensis and An. funestus are equally important vectors of malaria in Matola coastal suburb of Maputo, southern Mozambique Med Vet Entomol 2000 14 171 180 10.1046/j.1365-2915.2000.00228.x 10872861

[B58] Muriu SM Muturi EJ Shililu JI Mbogo CM Mwangangi JM Jacob BG Irungu LW Mukabana RW Githure JI Novak RJ Host choice and multiple blood feeding behaviour of malaria vectors and other anophelines in Mwea rice scheme, Kenya Malar J 2008 7 43 10.1186/1475-2875-7-43 18312667 PMC2291060

[B59] Midega JT Smith DL Olotu A Mwangangi JM Nzovu JG Wambua J Nyangweso G Mbogo CM Christophides GK Marsh K Bejon P Wind direction and proximity to larval sites determines malaria risk in Kilifi District in Kenya Nat Commun 2012 3 674 610.1038/ncomms1672 22334077 10.1038/ncomms1672 PMC3292715

[B60] Gu W Novak RJ Habitat-based modeling of impacts of mosquito larval interventions on entomological rates, incidence, and prevalence of malaria AmJTrop Med Hyg 2005 73 546 552 16172479

[B61] Gu W Regens JL Beier JC Novak RJ Source reduction of mosquito larval habitats has unexpected consequences on malaria transmission Proc Natl Acad Sci USA 2006 103 17560 17563 10.1073/pnas.0608452103 17085587 PMC1634411

[B62] Protopopoff N Bortel WV Marcotty T Herp MV Maes P Baza D D’Alessandro U Coosemans M Spatial targeted vector control in the highlands of Burundi and its impact on Malaria transmission Malar J 2007 6 158 10.1186/1475-2875-6-158 18053166 PMC2217530

[B63] Protopopoff N Bortel WV Marcotty T Herp MV Maes P Baza D D’Alessandro U Coosemans M Spatial targeted vector control is able to reduce malaria prevalence in the Highlands of Burundi AmJTrop Med Hyg 2008 79 12 18 18606758

[B64] Koekemoer LL Kamau L Hunt RH Coetzee M A cocktail polymerase chain reaction (PCR) assay to identify the Anopheles funestus (Diptera: Culicidae) group AmJTrop Med Hyg 2002 6 78 83 10.4269/ajtmh.2002.66.804 12224596

[B65] Beier JC Keating J Githure JG Macdonald MB Impoinvil DE Novak RJ Integrated vector management for malaria control Malar J 2008 7 Suppl 1 S4 10.1186/1475-2875-7-S1-S4 19091038 PMC2604879

[B66] WHO Global Strategic Framework for Integrated Vector Management 2004 Geneva: World Health Organization Document WHO/CDS/CPE/PVC/2004.10

[B67] WHO Handbook for Integrated Vector Management (IVM) 2012 Geneva: WHO Press WHO/HTM/NTD/VEM/2012.3

